# 

**DOI:** 10.1192/bjb.2023.95

**Published:** 2024-06

**Authors:** Brendan D. Kelly

**Affiliations:** Professor of Psychiatry in the Faculty of Health Sciences, Trinity College Dublin, and a consultant psychiatrist with Trinity Centre for Health Sciences, Tallaght University Hospital, Dublin, Ireland. Email: brendan.kelly@tcd.ie

**Figure:**
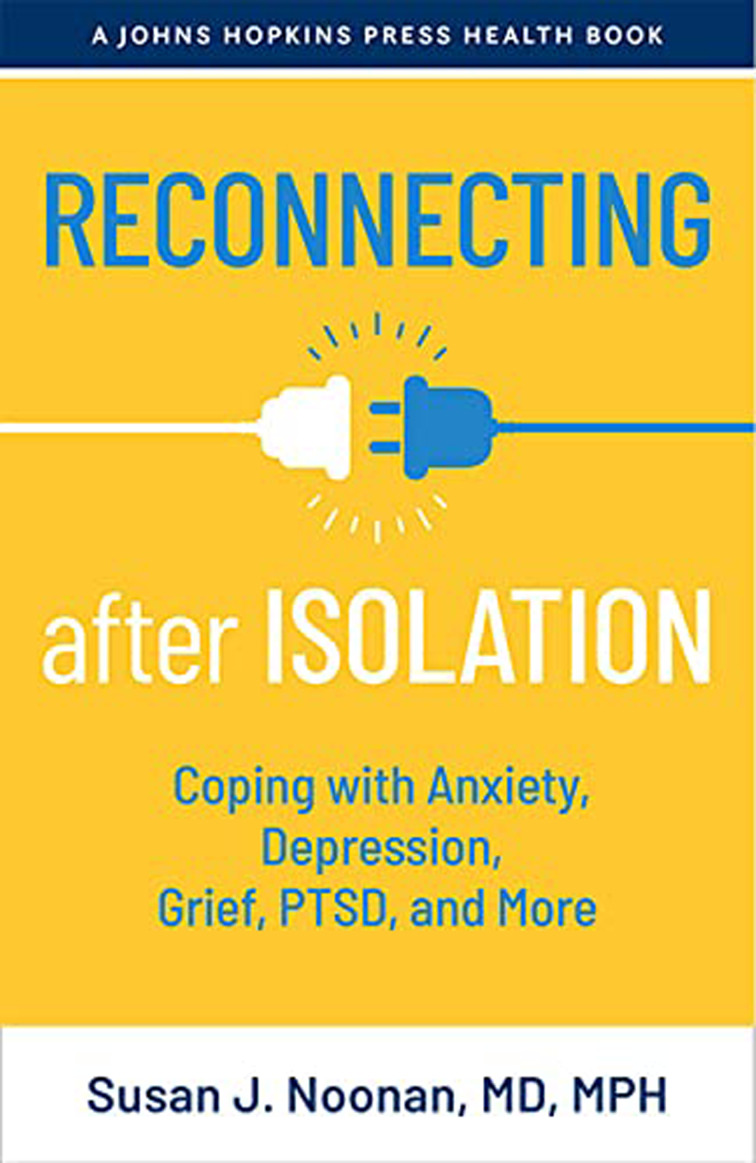

For many people, COVID-19 changed how they see themselves in the world. The pandemic brought a great deal of loss, uncertainty, isolation and grief. Sometimes, it felt as if the waves of infection would never stop, the lockdowns would never end.

These events presented real challenges, but they were not the whole story. Detailed statistics tell a nuanced tale of weaknesses and strengths, suffering and support. Against this rather complicated background, there is a need for mental health literature that highlights both problems and solutions associated with COVID-19, and issues that arise when societies open up as well as when they shut down.

Dr Susan J. Noonan's new book, *Reconnecting after Isolation: Coping with Anxiety, Depression, Grief, PTSD, and More*, meets many of these needs. The book is divided into two sections. The first section asks: ‘How does social isolation affect me?’ This covers such topics as social isolation, stress, coping skills, facing fears, fatigue, burnout, grieving, isolation, mental health, and substance misuse and addictions. There is an especially useful chapter about suicidal thoughts or impulses, which are commonly ignored in certain publications but are dealt with directly here. Noonan offers practical advice about professional care, risk reduction and, ‘most important’, providing ‘support and a strong connection’. Her approach to this topic reflects a valuable aspect of this book: its combination of solid professional advice with an intensely human approach to each individual.

The second section of the book asks: ‘What can I do to help myself?’ This explores the basics of mental health, finding effective care, talk therapy, resilience and ‘reentry anxiety’. The chapter titled ‘Is talk therapy right for me?’ is especially useful. Its explanation of cognitive–behavioural therapy has relevance beyond the post-COVID-19 context and will be useful to many people who are considering psychological care for this or other reasons.

Dr Noonan is well placed to address these themes. She is a physician, certified peer specialist, mental health and wellness coach, and consultant to Massachusetts General Hospital, McLean Hospital and the Depression and Bipolar Support Alliance. She is author of *Take Control of Your Depression: Strategies to Help You Feel Better Now* and *Helping Others with Depression: Words to Say, Things to Do*.

Dr Noonan's professional and writing experience are in clear evidence in *Reconnecting after Isolation*. This new book is a useful guide for people who are struggling to reconnect after periods of isolation, not only after the COVID-19 pandemic, but also following isolation for other reasons.

